# Effect of Propofol in the Immature Rat Brain on Short- and Long-Term Neurodevelopmental Outcome

**DOI:** 10.1371/journal.pone.0064480

**Published:** 2013-05-30

**Authors:** Tanja Karen, Gerald W. Schlager, Ivo Bendix, Marco Sifringer, Ralf Herrmann, Christos Pantazis, David Enot, Matthias Keller, Thoralf Kerner, Ursula Felderhoff-Mueser

**Affiliations:** 1 Department of Paediatrics I, Neonatology, University Hospital Essen, University Duisburg-Essen, Essen, Germany; 2 Department of Anaesthesiology and Intensive Care Medicine Berlin, Charité-Universitätsmedizin Berlin, Berlin, Germany; 3 Department of Anaesthesiology and Intensive Care Medicine, Asklepios Klinik Harburg, Hamburg, Germany; Hôpital Robert Debré, France

## Abstract

**Background:**

Propofol is commonly used as sedative in newborns and children. Recent experimental studies led to contradictory results, revealing neurodegenerative or neuroprotective properties of propofol on the developing brain. We investigated neurodevelopmental short- and long-term effects of neonatal propofol treatment.

**Methods:**

6-day-old Wistar rats (P6), randomised in two groups, received repeated intraperitoneal injections (0, 90, 180 min) of 30 mg/kg propofol or normal saline and sacrificed 6, 12 and 24 hrs following the first injection. Cortical and thalamic areas were analysed by Western blot and quantitative real-time PCR (qRT-PCR) for expression of apoptotic and neurotrophin-dependent signalling pathways. Long-term effects were assessed by Open-field and Novel-Object-Recognition at P30 and P120.

**Results:**

Western blot analyses revealed a transient increase of activated caspase-3 in cortical, and a reduction of active mitogen-activated protein kinases (ERK1/2, AKT) in cortical and thalamic areas. qRT-PCR analyses showed a down-regulation of neurotrophic factors (BDNF, NGF, NT-3) in cortical and thalamic regions. Minor impairment in locomotive activity was observed in propofol treated adolescent animals at P30. Memory or anxiety were not impaired at any time point.

**Conclusion:**

Exposing the neonatal rat brain to propofol induces acute neurotrophic imbalance and neuroapoptosis in a region- and time-specific manner and minor behavioural changes in adolescent animals.

## Introduction

Propofol (6,2 Diisopropylphenol) is widely used in paediatric anaesthesia. In 1999 the US Federal Drug administration decreased the approved age for maintenance of anaesthesia with propofol to 2 months, whereas in Germany the use of propofol 1% for induction and maintenance of anaesthesia is approved for children older than 1 month [1]. Despite these off-label restrictions propofol administration is common for anaesthesia in newborns and young children, even in preterms. In a recently published Cochrane review about propofol use in neonates only one open-label randomised controlled trial of 63 neonates was eligible for inclusion. Therefore no practice recommendation can be made based on the available evidence regarding the use of propofol in neonates [2]. Data on long-term neurological outcome in children after propofol administration are missing.

Positive attributes of propofol are its pharmacokinetic properties that account for its clinical benefits such as rapid onset and of anaesthesia and short recovery time.

Experimental studies revealed several mechanisms of action, which strongly depend on age of animals but also on the dose administered. Propofol potentiates GABA_A_ receptor functioning while at higher concentrations it causes opening of GABA_A_ receptors [3].

In adults propofol has been suggested as an ideal anaesthetic for neurosurgery because of its presumed beneficial effects on cerebral physiology (reduction in cerebral metabolic rate, reduction in cerebral blood flow, and brain relaxation). Experimental investigations revealed that propofol might also protect the brain against ischemic injury [4]–[6]. These neuroprotective effects of propofol have been attributed to its antioxidant properties, exponentiation of GABA_A_-mediated inhibition of synaptic transmission, and glutamate release [7]–[9].

However, in the immature brain general anaesthetics can induce apoptotic cell death in the central nervous system of experimental animals when administered during synaptogenesis that occurs during the first 2 weeks of life [10]. The apoptotic effect of propofol is age-dependent and coincidences with the vulnerability period to the pro-apoptotic effect of N-methyl-D-aspartate antagonists (NMDA), GABA_A_ agonists, sodium channel blockers, and ethanol [11]–[16].

Expression patterns of neurotransmitter receptors such as GABA and NMDA differ significantly between the adult and the newborn brain. This is probably a major reason for the peak vulnerability of the immature brain in the developmental period of rapid synaptogenesis, also known as the brain growth spurt period [17], [18]. Therefore, cellular mechanisms of general anaesthetic agents on the postnatal developing brain are of upmost interest of research [19].

To address this issue, we studied the temporal and regional activity of apoptotic and anti-apoptotic proteins and furthermore changes in the expression of neurotrophins following propofol anaesthesia on the developing brain. The second aim was to elucidate the long-term consequences on spontaneous behaviour, learning and memory abilities and its influence on anxiety-like behaviour. We hypothesise that administration of propofol to the immature rat brain causes neuroapoptosis, leading to a neurobehavioural phenotype/deficit in adolescence and adulthood.

## Materials and Methods

### Animal Experiments

All animal experiments were approved and performed in accordance with the guidelines of the Charité-Universitätsmedizin Berlin, Germany and the University hospital Essen, Germany. Animal care and handling were conducted in accordance with the European guidelines for use of experimental animals by certified FELASA fellows (Federation of European Laboratory Animal Science Associations) and with permission of local welfare committees. In the present study we employed a previously characterised animal model [11], [16]. In short, six-day-old (P6) Wistar rat pups were randomised to one of two groups and treated with intraperitoneal applications of 3×30 mg/kg BW propofol at 0, 90 and 180 min or injections with normal saline as controls. To maintain body temperature and to prevent hypothermia, animals were placed in a heating device maintaining an environmental temperature of 37°C. During anaesthesia respiratory frequency and skin colour were observed to detect apnea and hypoxia. If bradypnea occurred, rats received a painful stimulus, if breathing did not restart or resuscitation efforts were necessary rats were excluded from further processing and analysis. In total, five propofol treated animals had to be excluded from the experiment due to futile resuscitation efforts. One propofol treated animal was found dead in its homecage on P8. 57 animals were included for further analysis. 37 animals were sacrificed for molecular studies 6 (n_control_ = 7, n_propofol_ = 6), 12 (n_control_ = 6, n_propofol_ = 6) and 24 hrs (n_control_ = 6, n_propofol_ = 6) after administration of substances. Two samples derived from animals sacrificed 6 hrs after administration of substances had to be discarded due to a technical error in the preparation for immunoblotting therefore 11 animals were analysed by immunoblotting (n_control_ = 6, n_propofol_ = 5). 20 animals (n_control_ = 12, n_propofol_ = 8) were raised for behavioural testing on P30 and P120.

### Tissue Preparation

For molecular studies animals were sacrificed 6, 12 and 24 hrs after administration of substances, by i.p. injection of 1.5 g/kg BW chloral hydrate, followed by transcardial perfusion with sterile phosphate buffered saline (PBS). Olfactory bulb and cerebellum were discarded from brain tissue, and cortex and thalamus were micro-dissected, snap frozen in liquid nitrogen, and stored at −80°C until further analysis.

### Molecular Studies

Molecular analyses were focused on changes in apoptotic signalling pathways (caspase-3 activation and the caspase-independent apoptosis-inducing factor (AIF)), changes in neurotrophin expression patterns (BDNF, NT-3, NGF), and neurotrophin-dependent signalling pathways (serine-threonine kinase AKT, extracellular signal-regulated kinase ERK1/2, and their phosphorylated forms p-AKT and p-ERK1/2).

#### Quantitative Real-Time PCR

RNA was extracted from cortical and thalamic brain fractions, using Trizol (Invitrogen, Darmstadt, Germany) according to the manufacturer’s recommendations. 1 µg of total RNA was treated with DNase I (Invitrogen) in a total volume of 10 µl and 5 µl (500 ng) of this batch was reverse transcribed with 200 U SuperScript II (Invitrogen) using 500 ng oligo-dT and 250 ng random hexamers. cDNA was diluted and BDNF (GenBank accession no.: NM_012513; forward: 5′-ATGCTCAGCAGTCAAGTGCCTTTGG-3′ and reverse: 5′- GCCGAACCCTCATAGACATGTTTGC-3′), NT-3 (GenBank accession no.: NM_031073; forward: 5′-AGTGTGTGACAGTGAGAGCCTGTGG-3′; reverse: 5′-GAGAGTTGCCGGTTTTGATCTCTCC-3′) and NGF (GenBank accession no.: XM_227525; forward: 5′-ACCCAAGCTCACCTCAGTGTCTGG-3′; reverse: 5′-CATTACGCTATGCACCTCAGAGTGG-3′) amplified in duplicates and three independent experiments in a StepOnePlus Real-Time PCR System (Applied Biosystems, Darmstadt, Germany) with Fast SYBR® Green Master Mix (Applied Biosystems) according to the manufacturer’s recommendations, using β-actin as endogenous control. PCR amplification was performed in 96-well optical reaction plates for 40 cycles, with each cycle at 94°C for 3 s and 60°C for 30 s. Fold change was calculated using the 2^−ΔΔCt^-method [20].

#### Immunoblotting

Snap-frozen tissue was homogenised in RIPA (radio-immuno-precipitation assay) buffer (1% NP40, 0.5% sodium deoxycholate, 0.1% SDS, 1 mM EGTA, 1 mM Na_3_VO_4_, 20 mM NaF, 0.5 mM DTT, 1 mM PMSF and protease inhibitor cocktail in PBS pH 7.4). The homogenate was centrifuged at 1,050 g (4°C) for 10 min, and the microsomal fraction was subsequently centrifuged at 17,000 g (4°C) for 20 min. Twenty micrograms of the resulting cytosolic protein extracts were heat denaturated in Laemmli sample loading buffer, separated by electrophoresis in 10 or 15% SDS polyacrylamide gels and electro transferred onto a nitrocellulose membrane. Equal loading and transfer of proteins was confirmed by staining the membranes with Ponceau S solution (Fluka, Buchs, Switzerland). Nonspecific protein binding was prevented by treating the membrane with block solution (5% skim-milk, 0.5% BSA in TBST) 1 h at room temperature. The following primary antibodies (Cell Signaling, New England Biolabs GmbH, Frankfurt, Germany) were used for overnight incubation at 4°C: extracellular signal regulated kinase (ERK1/2, rabbit polyclonal p44/42 ERK 1/2, 1∶1000; mouse monoclonal phospho-p44/42 ERK1/2, 1∶500), protein kinase B (AKT, rabbit polyclonal AKT, 1∶1000; rabbit polyclonal phospho-AKT, 1∶1000), cleaved caspase-3 (rabbit polyclonal cleaved caspase-3, 1∶1000) and apoptosis-inducing factor (AIF, rabbit polyclonal AIF, 1∶1000). Primary antibodies were detected with appropriate horseradish-peroxidase labelled secondary antibody (swine anti-rabbit and rabbit anti-mouse 1∶2000-1∶10000, DAKO, Hamburg, Germany) and visualised using enhanced chemiluminescence (ECL, Amersham Biosciences, GE Healthcare, Buckinghamshire, UK). Serial exposures were made to radiographic film (Hyperfilm ECL; Amersham Biosciences, GE Healthcare). Densitometric analysis was performed with the image analysis program BioDocAnalyze (Whatman Biometra, Göttingen, Germany). For stripping, membranes were incubated with stripping buffer (100 mM β-mercaptoethanol, 2% SDS, 62.5 mM Tris-HCl; pH 6.7) at 50°C for 30 min, then washed, blocked and reprobed overnight at 4°C with mouse anti-β-actin monoclonal antibody (1∶1000, SIGMA-ALDRICH, Schnelldorf, Germany).

### Behavioural Studies

To assess the effect of propofol treatment on functional long-term outcome we performed behavioural testing on adolescent animals at postnatal day 30 and re-tested them at adult age on postnatal day 120. We focused on Open-Field test (OF) [21] and tests assessing activity and anxiety, Novel-Object-Recognition test (NOR) [22] assessing memory function. Tests were performed as described previously [23] with some modifications. In short, animals were handled, every other day, during their active phase (i.e. P23 or P113) to familiarise with the investigator one week before start of behavioural testing. Afterwards testing started with four days of OF followed by two days of NOR. All experiments were conducted one hour after lights out (18∶00 CET; active phase). Between tests, urine and defecation were removed and the arena was wiped clean with 50% 2-isopropyl alcohol. All trials were measured using an automatic tracking system (TSE Systems, Bad Homburg, Germany), and exported for data processing and statistical analysis.

#### Open field-Test

Animals were placed into the centre of a dimly lit open-field arena (51×51×40 cm), fabricated of biologically inert, infrared (IR) translucent material, placed upon a IR light-box (TSE Systems) emitting IR light (λ∼850 nm). Movements were tracked by an automatic monitoring system (VideoMot2, TSE Systems) for 5 min, the movement threshold was set to 0.5 cm. Activity parameters the time in motion (locomotion), travelled distance (distance) and speed were analysed. Anxiety was calculated as the percentage of time the animal stayed in the border areas of the box in relation to the total time spent in maze. This procedure was repeated every 24 hrs on four consecutive days in order to capture time dependent changes and as training for the NOR.

#### Novel Object Recognition Test (NOR)

Following the OF, we performed the NOR test, a two-trial, non-spatial, non-aversive memory test consisting of a “sample” phase and a “choice” phase, separated by a “test-free” interval during which the animals are returned to their home cages [22]. We analysed “test-free” intervals of 6 and 24 hrs. Two identical objects were presented for 10 min during the “sample” phase. In the “choice” phase, one object was replaced and the animals allowed to explore both objects for five minutes. All objects were made of biologically inert material. The NOR-task was analysed by automatic video tracking (VideoMot2, TSE Systems), using "three point detection", which distinguishes between the head, tail and centre of gravity. An encounter was defined as direct interaction with the object. Thus, rearing or sitting on the object was not valued as such. An animal spending more time with the novel object demonstrates its ability to remember the familiar object [24], reflected by the discrimination index [25] where positive values describe a preference for the novel object.

### Statistical Analysis

Data analysis and representations were performed within the statistical environment R [26]. Molecular analysis data were normalised to control, and reported as such for graphical representation. Statistical analyses of molecular analysis data were performed on pre-processed, log-basis 2 transformed data and reported as such without back-transformation. Log-basis 2 transformed fold change (log2FC) was used in the text to report treatment effects at single time-point and Δlog2FC for treatment effect differences between two sampling points/conditions. For assessment of anxiety, all analyses were performed on arcsine transformed data and also reported as such without back transformation. Several specifications of the error term were allowed to include the longitudinal structure (i.e. compound symmetry correlation), and to deal with potential time-dependent heteroscedasticity. P-values from post-hoc tests were adjusted according to the Holm-Bonferroni method [27] to account for multiple comparisons (expressed as q in the text). Model selection followed the protocols published in Zuur et al. [28]. Initial model formulation followed strictly the experimental design including treatment and trial as the main effects and interaction terms reflecting time×treatment dependent changes. This full model was further reduced by dropping non significant interaction terms. Fixed effects under consideration were tested with Wald tests. Unless stated, all values are reported and graphed as mean ± standard error (SE).

## Results

### Propofol Induces Activation of Caspase-3

Western blot analysis of brain lysates of cortex and thalamus at 6, 12 and 24 hrs after intraperitoneal applications of 3×30 mg/kg BW propofol at 0, 90 and 180 min in P6 rats, showed that propofol induces apoptotic neurodegeneration.

**Figure 1:**
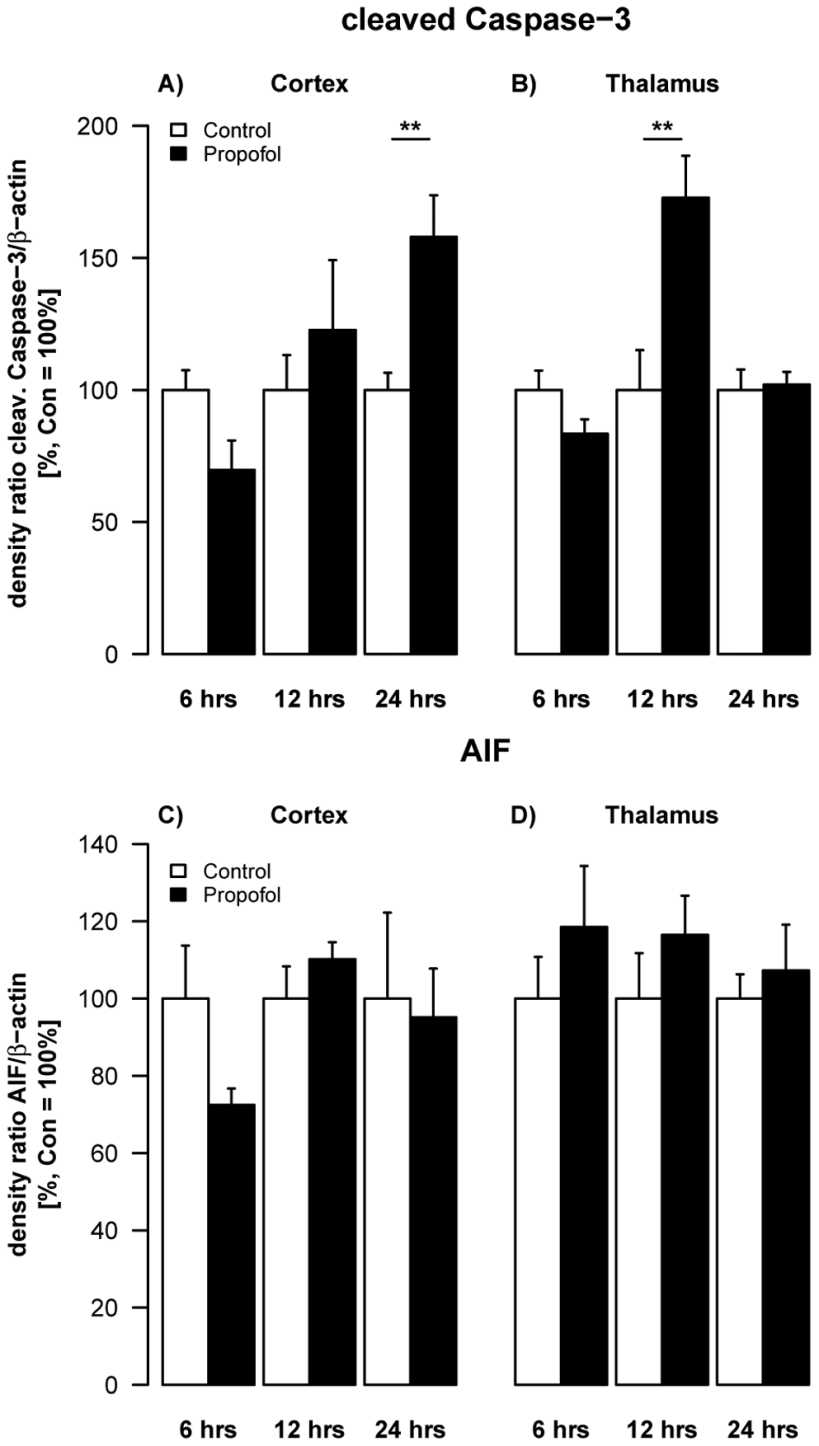
Impact of propofol on key proteins involved in apoptotic signalling. Densitometric quantifications of caspase-3 and AIF in cortex and thalamus of P6 rats as analysed by Western blotting. Values represent mean normalised ratios of the densities of caspase-3 and AIF bands compared to densities of the control group (n = 5–6/point+SE). There was an effect of propofol treatment on caspase-3 levels over time, which was significant after 24 hrs in the cortex [F(1,29) = 3.63, p = 0.06] and after 12 hrs in the thalamus [F(1,28) = 3.1, p = 0.09).

Propofol induced time dependent changes in the activation of caspase-3 in cortical (F(2,29) = 4.32, p = 0.023) and thalamic (F(2,28) = 6.61, p = 0.005) areas. The average difference of cleaved caspase-3 in cortical areas between treatment groups changed significantly from 6 to 24 hrs after the injection (Δlog2FC_(6 h: 24 h)_ = 1.22, SE = 0.42, t(29) = 2.92, q = 0.02). In thalamic brain regions we observed a significant increase in cleaved caspase-3 12 hrs after the last injection (log2FC_(12 h)_ = 0.83, SE = 0.26, t(28) = 2.92, q = 0.01). We further detected a significant change in the average difference between the two treatment groups from 6 to 12 hrs (Δlog2FC_(6 h: 12 h)_ = 1.08, SE = 0.30, t(28) = 3.63, q = 0.003) and 12 to 24 hrs (Δlog2FC_ (12 h: 24 h)_ = −0.79, SE = 0.29, t(28) = −2.73, q = 0.02). There was no significant effect on activation of AIF (Fig. 1A–D).

### Propofol Leads to Down-regulation of Neurotrophin mRNA in the Developing Rat Brain

**Figure 2:**
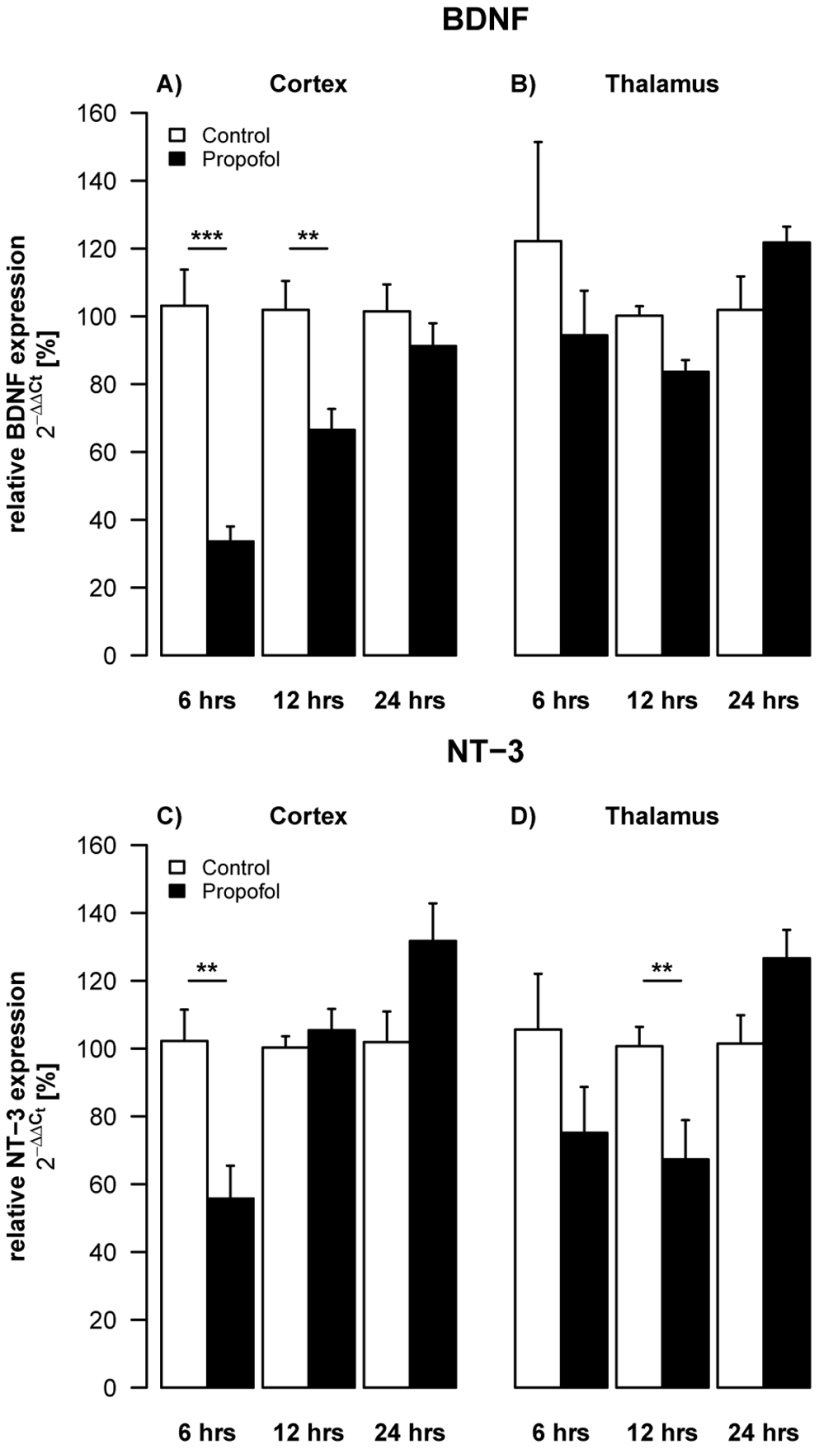
Impact of propofol on neurotrophins. Densitometric quantifications of mRNA levels of BDNF and NT-3 in cortex and thalamus of P6 rats, analysed by qRT-PCR. Values represent mean normalised ratios of the densities of BDNF and NT-3 bands compared to the density of the control group (n = 6–7/point+SE). There was an effect of propofol treatment with a decrease of BDNF levels over time, which was significant after 6 hrs in the cortex [F(1,30) = 66.5, p<0.001]. There was also a decrease in NT-3 levels, which was significant in the cortex after 6 hrs [F(1,28) = 12.7, p = 0.004] and after 12 hrs in the thalamus [F(1,24) = 3.5, p = 0.06].

To explore potential mechanisms involved in pathogenesis of apoptotic neurodegeneration in the developing brain following propofol exposure, we investigated whether expression patterns of brain-derived neurotrophic factor (BDNF), neurotrophin-3 (NT-3) and nerve growth factor (NGF) in cortex and thalamus of P6 rats (Fig. 2 A–D).

Propofol triggered an overall reduction (log2FC_(6 h–24 h)_ = −0.80, SE = 0.12, t(30) = −6.92, p = 1.11×10^−7^; Fig. 2A) of BDNF mRNA in cortical areas during the observed time frame from 6–24 hrs (F(1,30) = 66.5, p = 4.22×10^−9^; Fig. 2A). BDNF mRNA expression changed significantly over time (F(2,30) = 14.3, p = 4.28×10^−5^; Fig. 2A), with a significant decrease 6 hrs (log2FC_(6 h)_ = −1.64, SE = 0.20, t(30) = −8.15, q = 1.27×10^−8^) and 12 hrs (log2FC_(12 h)_ = −0.62, SE = 0.20, t(30) = −3.08, q = 8.83×10^−3^; Fig. 2A) but not 24 hrs (log2FC_(24 h)_ = −0.15, SE = 0.20, t(30) = −0.75 p = 0.46; Fig. 2A) after the last treatment. The average difference between the two treatment groups was significantly reduced from 6–12 hrs (Δlog2FC_(6 h: 12 h)_ = −1.02, SE = 0.28, t(30) = −3.59, q = 2.3×10^−3^; Fig. 2A) and 6–24 hrs (Δlog2FC_(6 h: 24 h)_ = −1.487, SE = 0.28, t(30) = −5.23, q = 3.6×10^−5^; Fig. 2A).

In thalamic areas BDNF mRNA expression of propofol treated animals changed significantly over time (F(2,24) = 5.21, p = 0.013; Fig. 2B). Whereas BDNF levels were not altered 6 hrs after the last injection we observed a significant reduction at 12 hrs (log2FC_(12 h)_ = −0.262, SE = 0.079, t(24) = 3.645, q = 0.004; Fig. 2B), with a significant increase during the following 12 hrs (Δlog2FC_(12 h: 24 h)_ = 0.542, SE = 0.168, t(24) = 3.228, q = 0.011; Fig. 2B). After 24 hrs levels of BDNF mRNA were restored and not significantly different from control animals.

Propofol treatment led to a significant, time dependent reduction of cortical NT-3 mRNA levels (F(2,28) = 8.45, 1.3×10^−3^; Fig. 2C). NT-3 expression was significantly decreased after 6 hrs (log2FC_(6 h)_ = −0.92, SE = 0.28, t(28) = −3.56, p = 4.02×10^−3^; Fig. 2C) but found to be restored 6 hrs later (log2FC_(12 h)_ = 0.06, SE = 0.10, t(28) = 1.98, p = 0.55; Fig. 2C) and did not significantly differ from controls at 24 hrs after the last injection (log2FC_(24 h)_ = 0.37, SE = 0.19, t(28) = 1.976, p = 0.12; Fig. 2C). Propofol administration also triggered a time dependent change of NT-3 mRNA (F(2,24) = 6.33, p = 0.006; Fig. 2D) in thalamic brain regions. Although we did not observe significant changes at any of the individual time points, we report a significant increase in the expression of NT-3 mRNA from 12 to 24 hrs following administration (Δlog2FC_(12 h: 24 h)_ = 0.986, SE = 0.308, t(24) = 3.204, q = 0.011; Fig. 2D).

Expression of NGF was transiently up-regulated in cortical areas at 6 hrs (log2FC_(6 h)_ = 0.71, SE = 0.19, t(30) = 3.82, p = 1.68×10^−3^) after the last injection of propofol, followed by a significant down-regulation after 12 and 24 hrs. In thalamic areas we observed a general down regulation (F(1.26) = 16.93, p = 3×10^−4^) of NGF mRNA over all time points (log2FC_(6 h–12 h)_ = −0.25, SE = 0.06, t(26) = −4.11, p = 3×10^−4^) (data not shown).

### Propofol Causes Regulation of the Active, Phosphorylated Isoforms of AKT and ERK1/2

**Figure 3:**
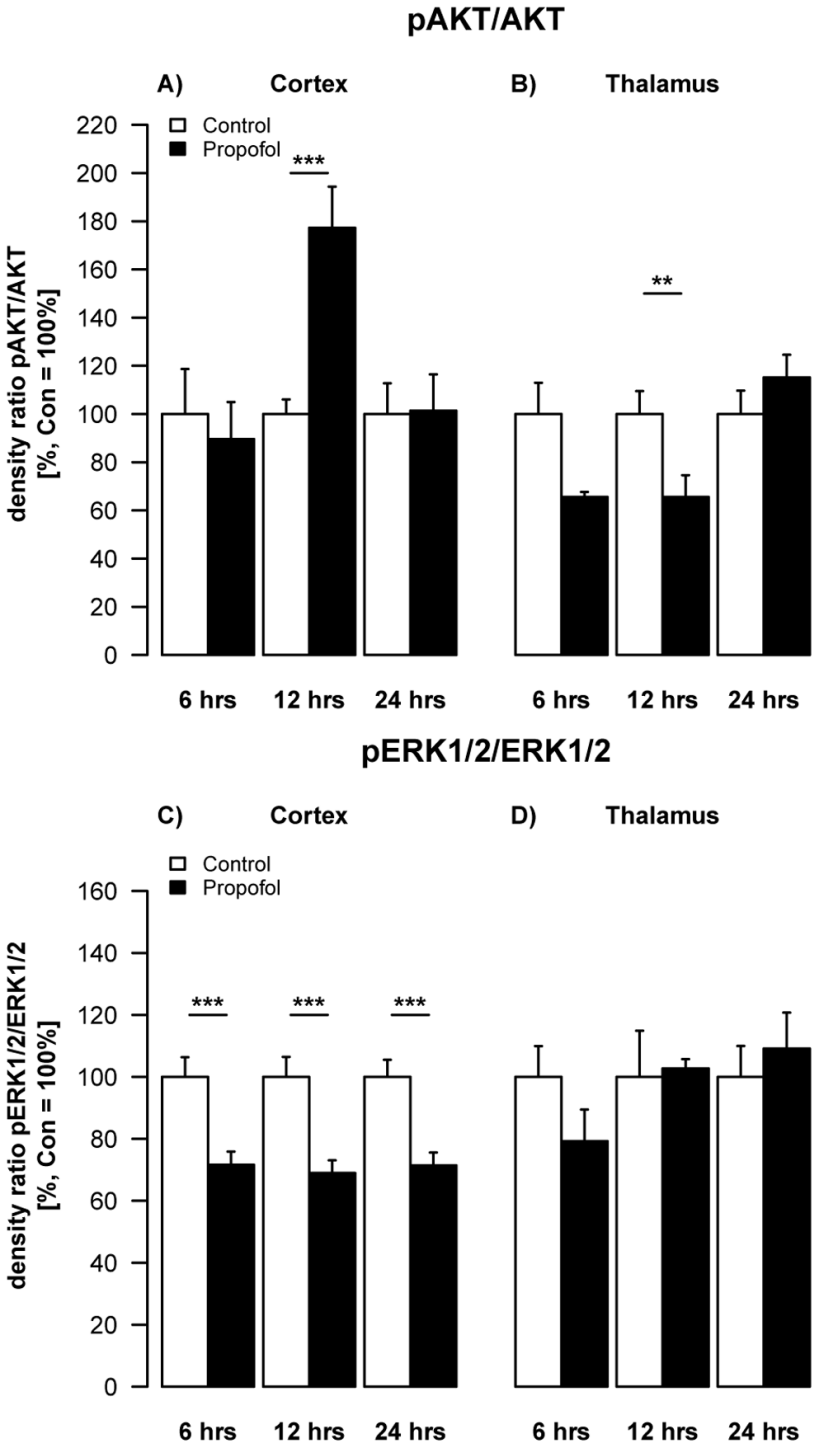
Impact of propofol on survival promoting proteins. Densitometric quantifications of pAKT and pERK1/2 in the cortex and thalamus of P6 rats, analysed by Western blotting. Values represent mean normalised ratios of the densities of pAKT and pERK1/2 bands compared to the density of the control group (n = 6/point+SE). There was an effect of propofol treatment in decrease of pAKT levels over time in the thalamus, which was significant after 12 hrs [F(1,28) = 5.6, p = 0.06]. Post-hoc analysis showed most pronounced decrease after 12 hrs (2-sample t-test). In the cortex there was a significant decrease of pERK1/2 levels over the time, which was significant after 6, 12 and 24 hrs [F(1,29) = 12.7, p = 0.013].

In the following step we investigated the influence of repeated propofol administration on the phosphorylated isoforms of the kinases AKT and ERK1/2. Western blot analysis of pAKT/AKT expression at 6, 12 and 24 hrs post injection, revealed a time dependent regulation of pAKT, in cortical (F(2,29) = 4.48, p = 0.02) and thalamic (F(2,28) = 4.49, p = 0.02) regions compared controls. The levels of pAKT were found to be significantly up-regulated in cortical areas after 12 hrs (log2FC_(12 h)_ = 0.81, SE = 0.16, t(29) = 5.51, q = 5.01×10^−5^; Fig. 3A), whereas the levels were significantly reduced in thalamic areas (Δlog2FC_(12 h)_ = 0.67, SE = 0.21, t(28) = 2.88, q = 0.02; Fig. 3B). We demonstrate a significant overall reduction (log2FC_(6 h–24 h)_ = −0.50, SE = 0.07, t(31) = −6.91, p = 9.37×10^−8^) in pERK1/2 expression on all three time points, (F(1,31) = 47.8, p = 9.37×10^−8^; Fig. 3C) in cortical regions. In contrast, thalamic levels in pERK1/2 expression were not significantly affected by propofol treatment (F(1,31) = 0.03, p = 0.8751; Fig. 3D).

### Propofol Treatment Leads to Increased Activity in Adolescent but not in Adult Animals

To assess the impact of these time- and region-dependent changes in neurodegeneration and neurotrophin-dependent signalling we investigated long-term behavioural and neurocognitive outcome longitudinal behavioural outcome (OF and NOR) on P30 and P120.

**Figure 4:**
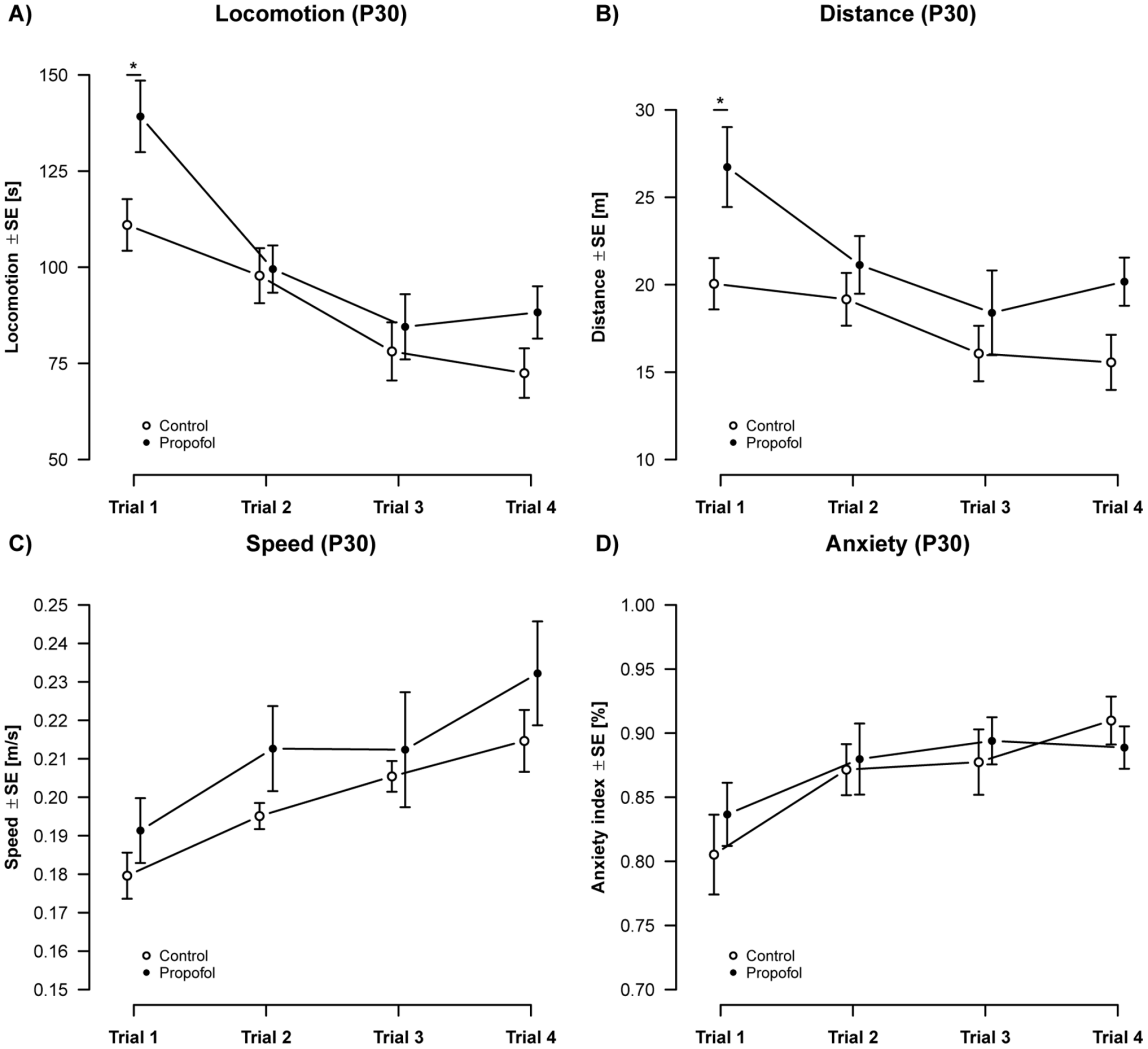
Activity (P30): Analysis of activity over 4 repeated measurements showed an overall increase in A) locomotion [F(1,71) = 7.12, p = 9×10^−3^] and B) distance [F(1,71) = 7.36, p = 8.37×10^−**3**^], but no change on C) speed [F(1,74) = 1.92, p = 1.69] in propofol treated animals. Propofol treatment did not alter D) anxiety related behavior in adolescent animals [F(1,74) = 0.02, p = 0.89]. An overall change in activity was observed over individual measurements, resulting in a significant decrease in locomotion [F(3,71) = 13.6, p = 4.08×10^−7^] and distance [F(3,71) = 5.35, p = 2.23×10^−3^] and a significant increase in speed [F(3,74) = 15.7, p = 5.53×10^−8^] and the index of anxiety [F(3,74) = 7.25, p = 3×10^−4^]. (n_controls_ = 12 animals, n_propofol_ = 8 animals).

Analysis of OF activity between P30 and P34 revealed an elevated pattern of general activity of propofol treated animals compared to controls (Fig. 4). Propofol treated animals spent more time in motion (12.8 s ±8.24 s SE; Fig. 4A), which was also reflected by an increased travel distance (3.87 m ±2.08 m SE; Fig. 4B). This increase was most pronounced on the first day of OF, where propofol treated animals travelled 28.2 s (±10.6 SE; t(71) = 2.67; q = 0.038) longer than control animals, and moved an additional distance of 6.67 m (±2.46 m SE) (t(71) = 2.71; q = 0.034). After the first day, observations in propofol treated animals dropped to levels comparable to the ones found in control animals. The average velocity of movements (speed) was not significantly different across groups (Fig. 4C). Animals of both treatment groups spent most of their time in the border area of the OF as expressed by the index of anxiety (AI). There was no significant difference between propofol treated and control animals (Fig. 4D), which indicates an undisturbed anxiety related behaviour in both groups.

**Figure 5:**
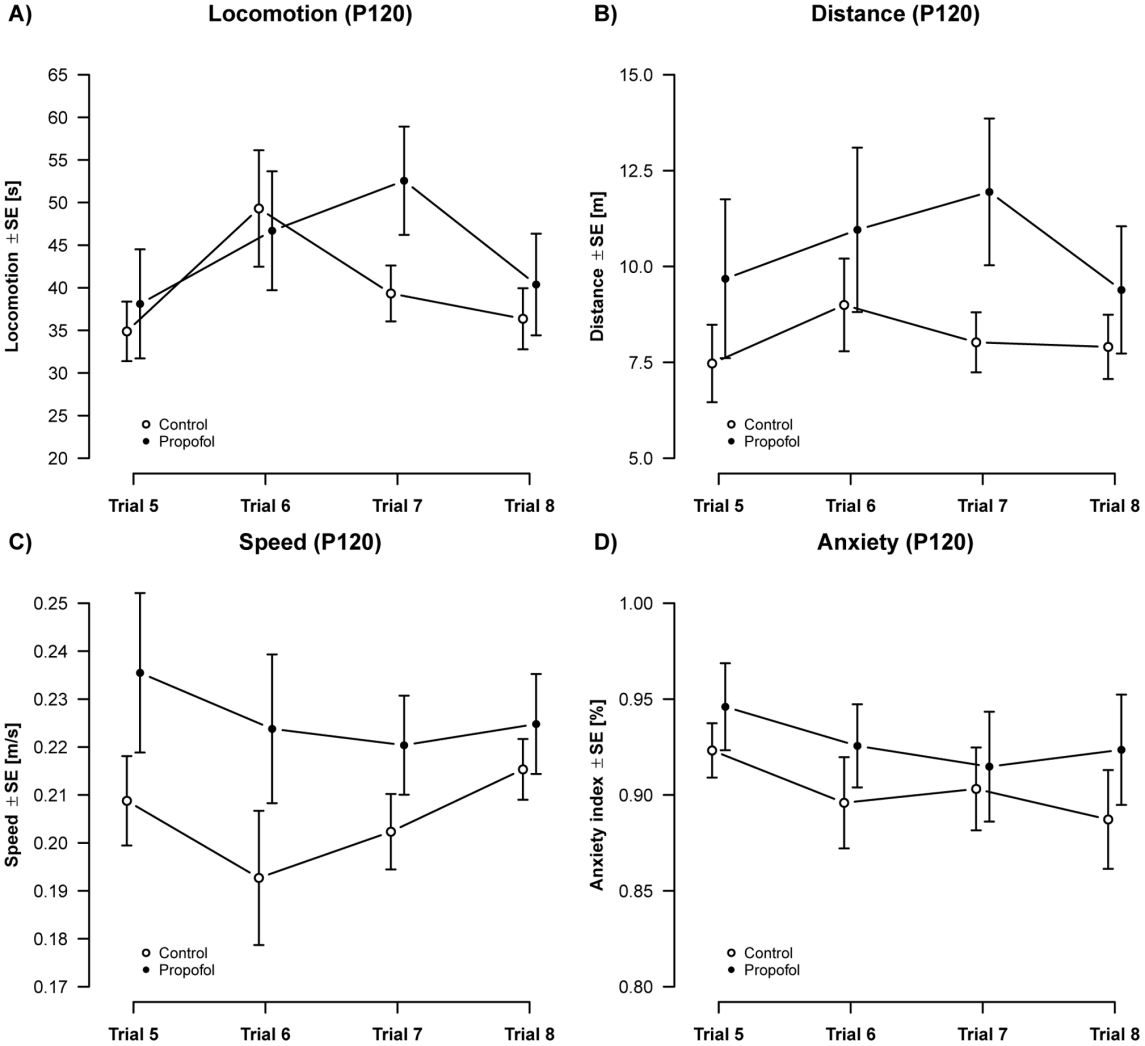
Activity (P120): Analysis of activity over 4 repeated measurements showed no treatment effects on A) locomotion [F(1,73) = 0.94, p = 0.33], B) distance [F(1,74) = 1.86, p = 0.18], C) speed [F(1,74) = 0.44, p = 0.51] or D) anxiety related behavior [F(1,62) = 0.57, p = 0.45] in propofol treated animals. Apart from a transient effect on locomotion [F(3,71) = 5.92, p = 1.13×10^−3^], no significant changes over repeated measurements were observed in adult aged animals. (n_controls_ = 12 animals, n_propofol_ = 8 animals).

Assessment of OF activity parameters between P120 and P124 (Fig. 5) showed, that alterations estimated in adolescent rats (P30) were not present in adult animals. There were no detectable changes in locomotion (Fig. 5A) and travel distance (Fig. 5B) as there were in P30 aged animals. The average velocity of movements (speed) did also not significantly differ between groups (Fig. 5C) in adult animals (P120). Similar to the estimations at P30 there were no significant changes in the AI in propofol treated and control animals (Fig. 5D), which again indicates an undisturbed anxiety related behaviour in both groups.

### Propofol Treatment has no Effect on Memory Performance

**Figure 6:**
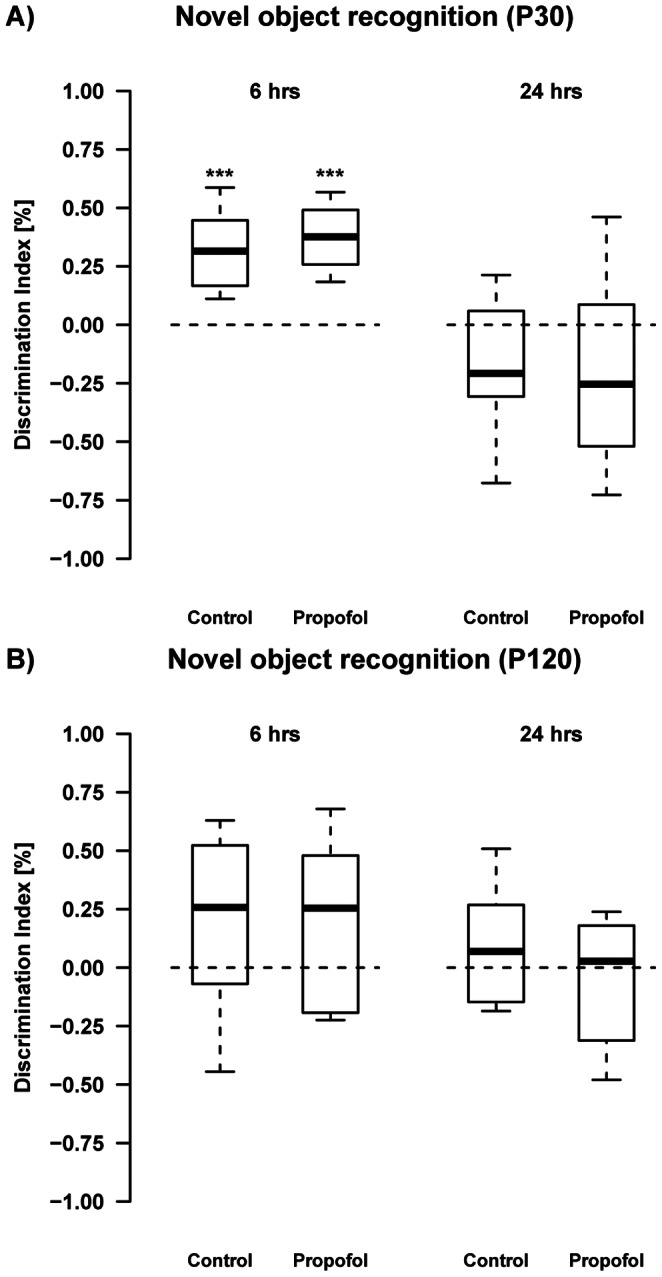
Novel object recognition on P30 and P120: At the age of 30 days, both propofol treated animals (t(7) = 7.45, ***q = 4.3×10^−4^) and controls (t(10) = 6.30, ***q = 3.6×10^−4^) spent significantly more time with the novel object indicating their ability to discriminate the novel from the old object. Propofol (t(7) = −1.44, q = 0.192) as well as control animals (t(10) = −1.92, q = 0.168) failed to do so after a 24 hrs inter-trial interval. At P120 both groups spent a random amount of time with either of the objects after 6 hrs and also after a 24 hrs interval, indicating that they were unable to remember the old object. (n_controls_ = 12 animals, n_propofol_ = 8 animals).

The assessment of cognitive performance in P30 animals (Fig. 6A) indicated that both, propofol treated animals and controls spent more time with a new object than with an old object, after a 6 hrs test-free interval. This indicates that both groups were able to remember the old project over the given time. After increasing the test-free interval to 24 hrs in the second trial, both, propofol treated as well as control animals, were no longer able to clearly discriminate between the new and the old object. In adult aged animals (P120, Fig. 6B) neither propofol treated animals, nor controls spent more time with the new than with the old object, which again indicates that both groups were not able to remember the old object. After increasing the test-free interval to 24 hrs in the second trail, there was also no indication that either propofol treated or control animals were able to remember the old project.

## Discussion

Based on the previously well described propofol-induced apoptotic neurodegeneration when administered in the first week of rodent life [11], [16], [29]–[34], the present study revealed a transient acute increase of apoptotic caspase-dependent signalling in combination with a down-regulation in neurotrophin-dependent proteins. These changes however did not lead to a distinct neurobehavioural phenotype/deficit in our assessment. Although we observed a significant increase in activity (locomotion and travel distance) on P30, both treatment groups showed similar behaviour in all other observed time points and parameters.

Our results indicate that apoptotic neurodegeneration was mainly mediated by caspase-3 activation, whereas there was no difference between experimental groups in expression of apoptosis-inducing factor (AIF). There were also spatial and time-dependent differences in caspase-3 expression with increased levels at 12 hrs in the thalamus, whereas the cortex was affected later (24 hrs), which is in accordance with previous observations of other groups [33], [34]. AIF is one of the key components of the caspase-independent cell death pathway [35], [36] and has been shown to be up-regulated by several noxious stimuli i.e. glutamate, oxidative stress, meningitis, trauma and ischemia [37]–[40]. Furthermore, studies following a hypoxic-ischemic insult to the developing brain indicated that AIF and caspases-1, -3, and -7 act through parallel pathways leading to neuronal death [41]–[43]. However, propofol treatment had no effect on this specific caspase-independent apoptotic pathway.

In this study we have chosen caspase-3 and AIF activation as representative markers for caspase-dependent and independent pathways to further characterise apoptosis in our animal model. These findings imply caspase involvement but do not exclude the possibility of many other death- and survival-promoting factors involved in the apoptotic machinery also contributing to propofol-induced acute injury in the immature brain.

Signalling pathways regulating cell death in development and after brain injury are not fully elucidated. Our findings indicate that propofol anaesthesia acutely depresses endogenous neurotrophins which have been shown to be crucial for neuronal development, synaptic plasticity and survival [44], [45]. Hyperoxia depressed synthesis of the neurotrophins BDNF, NT-3 and NGF and also reduced levels of the active phosphorylated forms of AKT and MAPK in cortex and thalamus in a time-dependent fashion [46], [47].

Such changes may reflect impairment of survival promoting signals resulting in an imbalance between neuroprotective and neurodestructive mechanisms, which, during a developmental period of ongoing physiological elimination of brain cells, can promote apoptotic death.

Recent studies have emphasised, that anaesthetics caused disturbances in neurotrophin homeostasis in the developing brain. General anaesthesia with midazolam, isoflurane and nitrous oxide caused a decrease of BDNF in thalamus and an increase in cortex [48], whereas propofol resulted in a decrease in NGF in thalamus and cortex [34], both with strong involvement of the AKT pathway [34], [48].

However, propofol anaesthesia in our model resulted in a significant reduction of neurotrophin availability (BDNF, NT-3, and NGF) in cortex and thalamus and significantly decreased activation of ERK1/2 in cortical areas at all time points investigated (6, 12 and 24 hrs). Prevention of cell death by the pERK1/2 pathway has been previously shown in cultured rat hippocampal neurons [49].

It remains unclear why the pERK1/2-pathway in our study was more affected than the pAKT-pathway. Based on presently available data, the variation in neurotrophin expression and function during development of each brain region is time-specific and may explain, at least in part, region-specific differences in an anaesthesia induced insult. Furthermore, our findings on reduced neurotrophin expression may explain anaesthesia-induced damage in the most vulnerable regions of the developing brain (thalamus and cortex) [15], [34], [48].

In addition, results addressing neurotrophin expression under pathological conditions in the immature brain strongly depend on the type of injury and the experimental model used. In traumatic injury to the developing brain, neurotrophin up-regulation has been observed [50], [51], whereas antiepileptic drugs and hyperoxia exposure down-regulate neurotrophins [12], [46], [47].

In order to determine long-term consequences of propofol we investigated whether the previously well-documented propofol induced neuroapoptosis in combination with an acute impairment of neurotrophin dependent signalling is reflected by sustained functional cognitive and motor impairment. A cumulative dose of 90 mg/kg BW i.p. propofol increased locomotive activity in 30 day-old adolescent animals, expressed by an increase in the time spent in motion, which led to an increased travel distance. The speed was not significantly altered between treatment groups, indicating that the observed changes were due to an increased locomotion, rather than speed. We suggest that the observed increased activity was triggered by the novel environment, since behaviour of experimental animals and control animals did not differ in the following days. This hypothesis is further supported by our observations from P120 to P124 which also revealed no alteration in these parameters.

Both treatment groups at P30 and P120 showed a similar change in parameters observed over repeated measurements. We therefore cannot conclude a significant inability to habituate to the testing procedure. This finding stands in contrast to the work from Bercker et al. [11] who showed a disturbance in the animal’s ability to habituate to the test procedure, from the first to the second day of the hole-board test, when treated with propofol. However, it has to be taken in to consideration that, Bercker et al. [11] used not only a different test for habituation, but also animals at seven weeks of age (P49). It is well known that results obtained from behavioural testing conducted in different laboratories can be substantially different even if the same tests and standardised protocols are enforced [52].

Fredriksson et al. [31] showed in a murine model (ten-day-old NMRI-mice), that neither the administration of 10 mg/kg nor 60 mg/kg BW propofol, on the tenth day after birth, resulted in a significant alteration of animal behaviour at P55.

Anaesthesia with propofol on P6 in our study did not result in memory deficits neither on P30 nor on P120, which is in accordance with previous results obtained by Bercker et al. [11] and Fredriksson et al. [31]. Even by using different tests, species and time points, none of the results indicate an impairment in memory functioning in propofol treated animals, which is in contrast to treatment with other clinically relevant anaesthetic drugs. A cocktail of midazolam, nitrous oxide, and isoflurane for 6 hrs caused widespread neurodegeneration in the developing brain and neurocognitive deficits that persisted throughout adolescence into adulthood [15]. This finding was explained by the authors that the anaesthesia-exposed rats had lasting deficits in hippocampal synaptic function and furthermore, memory functions are mediated by distributed network that includes, in addition to the hippocampus, anterior thalamic nuclei, mammillary bodies, and retrosplenial cortex. Each of the latter three structures was damaged in the anaesthesia-exposed brains more severely than the hippocampus [15].

Similar findings have been described after neonatal exposure to isoflurane alone [53] and propofol when the Morris water maze test was applied [11]. Fredriksson et al. [31], [54] exposed infant mice to the NMDA antagonist ketamine, or GABA agonists (diazepam, thiopental and propofol) and demonstrated that these drugs, especially if used in combination, can cause long-term locomotor and cognitive deficits. But interestingly no significant effects on spontaneous behaviour or habituation were seen when these mice were exposed to propofol or thiopental alone [31].

The present work suggests that acute propofol-induced neurodegeneration combined with a transient disturbance in neurotrophin availability observed in the thalamus and cortex has no long-term effects on cognitive performance in this model.

Upon the translation of our experimental results into the human situation several questions remain open. The first critical issue concerns the extrapolation of appropriate developmental stages from different animal species to humans [55]. The use of 6 day old rats in our study was based on the assumption that this neurodevelopmental age in the rat is equivalent the human brain to the period between week 25 of gestation and 1 year of age [17]. Another related concern is that, dosing paradigms used in animals studies typically do not reflect doses used for pediatric patients, and are 20–50 times higher in animals due to species dependent different metabolism [10]. Propofol-induced neurotoxicity depends on concentration and duration of treatment, as shown in *in vitro* studies, and on the dose, as revealed in *in vivo* studies in mice [29], [31], [56]. Here we used Propofol doses of 30 mg/kg body weight every 90 min up to a cumulative dose of 90 mg/kg. Doses of propofol were determined in pilot studies aiming at deep of anesthesia with no or only minor reaction to a pain stimulus while maintaining sufficient spontaneous breathing and normal skin color [11], [16]. The pharmacokinetic of propofol in human neonates remains relatively unexplained. A study of Allegaert et al. [57] explored the pharmacokinetics of a 3 mg/kg propofol bolus, followed by 8 mg/kg propofol for 1 hr, and a subsequent decrease to 6 mg/kg for another 1 hr, resulted in a significant higher (50–100%) propofol concentration in a preterm infant compared to a term newborn infant. Previous animal studies revealed that unopposed painful stimulation early in life can lead to neuronal cell death, as well as long-lasting imbalances of the immature nervous system, decreased pain thresholds and behavioural abnormalities later in life (58–60). The administration of propofol in our study was made by intraperitoneal injection. To exclude the effect of “painful” intraperitoneal injections on neuronal cell death the control group received sham injections at the same time points. However, there is no evidence in the literature that intraperitoneal injections alone may lead to a comparable increase in neurodegeneration. If bradypnea occured in our study, rats received one painful stimulus, if breathing did not restart or resuscitation efforts were necessary rats were excluded from further processing and analysis as previously described by our group [11], [16]. Also in preterm infants prolonged pain exposure alters their subsequent pain processing, long-term development and behaviour [61]. Furthermore, numerous procedures that have been associated with pain and stress in preterm infants are not accompanied by analgesia [62], [63]. In general, therapeutic, but also toxic effects of analgetics and anesthetics in the immature brain must be considered. Long-term effects of analgesic or anesthetic drugs depend on whether they are given in the presence or absence of painful stimulation [64], [65]. Ketamine anesthesia in neonatal rats during painful injections ameliorated the deleterious effects of painful stimulation, without causing neurodegenerative effects [64]. Thus, the effects of surgery without anesthesia and of anesthesia without surgery may be detrimental for the developing brain [65]. However, it is ethical unacceptable to subject infants to painful invasive procedures without the benefit of anesthesia and analgesia [66].

Therefore analgesic or anesthetic treatments should be tailored to the invasiveness or presumed pain intensity of the procedure [63].

The currently available experimental and clinical data addressing the toxic effects of anesthetics and sedatives and the impact of pain and stress on the developing brain are not sufficient and evidence-based enough to make any scientifically based recommendations for pediatric surgery or anesthesia [66], [67]. Despite all limitations, the effects of anesthesia and the stress response and/or inflammatory response of surgery on the patients well-being cannot be dimissed. To date no clinical trial investigated neurological sequelae after application of propofol or other anaesthetic regimens in the newborn.

In conclusion, exposure of propofol to the neonatal rat brain induces acute neurotrophic imbalance and neuroapoptosis in a region- and time specific-manner. In the long-term it resulted in minor behavioural changes in adolescent but not in adult animals. As clinical studies are still lacking, future research has to focus on the investigation of safe anaesthetic strategies possibly in combination with neuroprotective agents. Until then, caution should be taken when using anaesthetic agents alone or in combination in preterm infants, newborns and young children.
